# A study on the treatment of enamel white spot lesions using chitosan-calcium phosphate microgels loaded with EGCG-NPs

**DOI:** 10.3389/fphar.2026.1805278

**Published:** 2026-05-20

**Authors:** Yujia Zhang, Jinrui Liu, Kexin Yan, Xiaoyu Sun, Chenyang Niu, Donghong Yang

**Affiliations:** 1 Stomatology College of Jiamusi University, Jiamusi, China; 2 Department of Orthodontics, Affliated Stomatological Hospital of Jiamusi University, Jiamusi, China

**Keywords:** epigallocatechin-3-gallate (EGCG), hydrogel, orthodontics, remineralization, white spot lesions

## Abstract

**Background:**

During orthodontic treatment, fixed orthodontic appliances complicate optimal oral hygiene maintenance, frequently causing white spot lesions (WSLs). Clinically, WSLs are typical chalky-white opacities on the enamel surface, recognized as the early reversible stage of dental caries. In this study, a novel antibacterial and remineralizing material—CEN composites (Chitosan-Ca_3_(PO_4_)_2_, CS-CaP) microgels loaded with EGCG nanoparticles (NPs)—was designed to treat enamel white spot lesions.

**Methods:**

Material characterization experiments were conducted on EGCG-NPs, chitosan-calcium phosphate microgels and CEN composites; the dialysis bag diffusion method was used to determine the composite drug release rate. Enamel blocks were prepared, an acid-etched demineralization model was established, and *in vitro* remineralization experiments were performed to screen the optimal CEN composite concentration for enamel remineralization and confirm the synergistic effect of EGCG-NPs on remineralization. The CCK-8 method was employed to assess the cytotoxicity of the CEN complex.

**Conclusion:**

Material characterization experiments confirmed the successful synthesis of CEN composites. Drug release tests showed EGCG-NPs release rate reached 50% at 10 h and 80% at 70 h. *In vitro* remineralization experiments confirmed that the CEN-5 group was the optimal concentration for remineralization. *In vitro* experiments of the CEN-5 group demonstrated that EGCG-NPs significantly enhanced the gel’s remineralization efficacy. The CCK-8 assay demonstrated that the CEN composite possessed no cytotoxic effects.

## 1 Introduction

In recent years, advancements in living standards, coupled with continuous breakthroughs in orthodontic fundamental research and the increasing maturity of orthodontic techniques, have led to more refined aesthetic demands from patients. Beyond the pursuit of aligned teeth, there are now higher expectations for tooth surface smoothness and overall appearance. However, following orthodontic treatment, some patients develop chalky white spots on the labial and buccal surfaces of the teeth and around bracket areas, which compromise dental aesthetics. The primary reason for this phenomenon is that the presence of orthodontic appliances complicates oral hygiene, making it difficult to clean the teeth effectively and limiting oral self-cleaning ability. This environment facilitates the adherence and accumulation of dental plaque on the tooth surface, ultimately leading to the formation of these chalky lesions, known as white spot lesions (WSLs) (Sardana et al., 2023). These lesions are a relatively common complication during orthodontic treatment.

Studies report that the incidence of WSLs ranges from 23.4% to 75.6% (Julien et al., 2013; Sagarika et al., 2012). Although WSLs possess some potential for spontaneous recovery, they can persist for over 15 years post-treatment, with complete regression observed in only 28% of affected teeth (Bock et al., 2024). Therefore, the effective prevention and control of WSLs, along with promoting remineralization in affected areas, remain critical research directions in orthodontics.

Current treatment modalities for enamel demineralization include pharmacological approaches (e.g., fluoride preparations), laser therapy, resin infiltration, and remineralization therapy (Lin et al., 2025; Al-Blaihed et al., 2024). Among these, remineralizing agents are considered the first-line strategy for preventing and treating WSLs, as they can effectively halt the progression of white spot lesions towards cavitation (Hu et al., 2020). In recent years, by simulating the natural process of tooth development and mineralization, researchers have developed various nanomaterials that demonstrate excellent efficacy in repairing enamel defects (Yang M. et al., 2025; Pan et al., 2024). Furthermore, remineralization offers advantages such as non-invasiveness, good biocompatibility effects. Consequently, remineralization technology holds significant promise for providing a curative strategy for WSLs.

Chitosan (CS), a natural cationic polysaccharide, has garnered widespread attention in the biomedical field due to its low toxicity, excellent biocompatibility, biodegradability, immunomodulatory effects, and properties including anticancer, anti-injury, antioxidant, anti-inflammatory, and antimicrobial activities (Wang et al., 2023). Leveraging its exceptional chemical and biological characteristics, CS has been extensively utilized in the remineralization of enamel and dentin (Tang and Chen, 2022). The positive charges carried by CS enable it to bind to negatively charged *Streptococcus* mutans, thereby inhibiting its growth. Under low pH conditions, the positive charge of CS enhances its adhesion to the demineralized enamel surface, forming a physical barrier that reduces acid penetration and subsequently inhibits pathological demineralization (Pourhajibagher et al., 2022). In the context of enamel remineralization, CS is often employed in the form of hydrogels. Hydrogels offer clinical advantages such as ease of application and prolonged retention on the tooth surface, which can significantly enhance the remineralization repair effect.

Epigallocatechin gallate (EGCG), a polyphenolic monomer isolated and purified from tea, possesses a broad spectrum of biological activities. It exhibits antibacterial, antiviral, and antioxidant effects; it can also help prevent atherosclerosis, thrombosis, and angiogenesis by regulating relevant physiological processes. Furthermore, EGCG demonstrates anti-inflammatory and anticancer properties, along with relatively low biological toxicity (Capasso et al., 2025; Carneiro et al., 2016; Le et al., 2018). In dental research, EGCG has been found to inhibit dentin demineralization and promote its remineralization (Li et al., 2020). From a molecular structural perspective, the multiple hydroxyl functional groups in the EGCG molecule enable it to chelate metal ions. The combination of EGCG with calcium ions can form acid-resistant precipitates. These precipitates can potentially protect enamel and dentin from acid erosion, creating favorable conditions for the remineralization process, which is significant for preventing erosive tooth wear (Wang et al., 2018). However, the clinical translation of EGCG is severely limited by its low bioavailability. Its *in vivo* half-life is only approximately 3–5 h, and its stability is significantly affected by pH, decreasing in neutral environments and deteriorating further under highly alkaline conditions (Radhakrishnan et al., 2016). Nanoparticles have become a research focus in drug delivery systems due to their advantages, including strong tissue penetration, good colloidal stability, and the ability to enhance drug bioavailability. Encapsulating or loading EGCG into nanoparticles can effectively improve its stability and bioavailability.Based on this rationale, the present study designed and synthesized a chitosan-calcium phosphate-EGCG nanoparticle composite (CS-CaP-EGCG-NPs). Experimental characterizations verified the successful fabrication of the material. Furthermore, its *in vitro* remineralization potential and cytotoxicity were assessed, providing a theoretical reference for the management of enamel white spot lesions.

## 2 Methods

### 2.1 Material synthesis

#### 2.1.1 Synthesis of EGCG nanoparticles

EGCG nanoparticles (EGCG-NPs) were fabricated via the emulsion solvent evaporation technique. Briefly, 80 mg of glycerol monostearate, 20 mg of stearic acid, and 20 mg of soy lecithin were dissolved in dichloromethane, followed by sonication for 3 min. EGCG was dissolved in 200 µL of distilled water. A 2% (w/v) Pluronic F68 solution was added to the lipid phase to form a primary emulsion, which was then sonicated for 5 min. The primary emulsion was subsequently poured into a 1% (w/v) Pluronic F68 aqueous solution to generate a double emulsion. The obtained emulsion was magnetically stirred for 2 h. The resulting suspension was centrifuged, washed with distilled water, freeze-dried, ground, and stored for subsequent characterization and use (Li et al., 2024).

#### 2.1.2 Synthesis of chitosan-calcium phosphate microgels

Chitosan-calcium phosphate (CS-CaP) microgels were synthesized by ionic gelation. Chitosan (0.2 g and 0.15 g, respectively) was dissolved in 10 mL of 1% (v/v) acetic acid. The solution was stirred at 100 rpm, and the pH was adjusted to 4.76 with 0.1 mol/L KOH solution. Under the same conditions, 5 mL of 0.46 mol/L CaCl_2_ solution was added dropwise. Sodium tripolyphosphate (TPP) was blended with 5 mL of 0.46 mol/L K_2_HPO_4_ to prepare a TPP solution at a concentration of 0.29% (w/v). Subsequently, 10 mL of the TPP solution was added to the chitosan solution under continuous stirring at 500 rpm until a milky white suspension was obtained, indicating the successful formation of CS-CaP microgels (Simeonov et al., 2019).

#### 2.1.3 Synthesis of CS-CaP-EGCG-NPs composites

EGCG-NPs at concentrations of 30, 60, and 120 μg/mL were mixed with 1.5% and 2% CS-CaP microgels under sonication to fabricate chitosan-calcium phosphate-EGCG nanocomposites (abbreviated as CEN composites).

Six experimental groups were designed:

Group 1: 30 μg/mL EGCG-NPs + 1.5% CS-CaP
Group 2: 30 μg/mL EGCG-NPs + 2% CS-CaP
Group 3: 60 μg/mL EGCG-NPs + 1.5% CS-CaP
Group 4: 60 μg/mL EGCG-NPs + 2% CS-CaP
Group 5: 120 μg/mL EGCG-NPs + 1.5% CS-CaP
Group 6: 120 μg/mL EGCG-NPs + 2% CS-CaP

### 2.2 Material characterization

1. CS-CaP microgels, EGCG-NPs, and CEN composites were freeze-dried.
2. Morphological characterization: The freeze-dried CS-CaP microgels were cut into uniform specimens (5 mm × 5 mm × 2 mm^3^) using a sterile blade to ensure flat and intact surfaces. Morphological observations were performed using scanning electron microscopy (SEM).
3. Dynamic light scattering (DLS) measurements were conducted at 25 °C using a 35 mW red diode laser with a scattering angle of 90°. EGCG nanoparticle suspensions were placed in microplates for detection, and light scattering data were collected.
4. Zeta Potential Measurement: An appropriate amount of nanoparticles was dispersed in 1 mL of ultrapure water to prepare a dilute homogeneous suspension, followed by sonication for 3 min to ensure complete dispersion without visible precipitation. The EGCG-NPs suspension was slowly injected into the sample cell using a syringe for zeta potential analysis, and the results were recorded.
5. Fourier Transform Infrared Spectroscopy (FTIR): Freeze-dried powders of EGCG-NPs, CS-CaP, and CEN composites were mixed with potassium bromide (KBr), ground, and compressed into pellets. Fourier transform infrared (FTIR) spectra were recorded over the wavenumber range of 400–4,000 cm^-1^.
6. Drug loading efficiency and encapsulation efficiency of EGCG-NPs: 5 mg EGCG-NPs was dispersed in 1 mL dichloromethane, and EGCG was extracted using deionized water. The absorbance of the extract at 274 nm was determined using a UV-Vis spectrophotometer.The actual EGCG content was calculated from a pre-established calibration curve.
  Drug loading efficiency (%) = (Actual mass of EGCG in nanoparticles / Mass of EGCG- NPs) × 100%; Encapsulation efficiency (%) = (Actual mass of EGCG in nanoparticles / Total mass of EGCG added during preparation)×100%
7. *In Vitro* Drug Release: The *in vitro* drug release behavior of CEN composites was investigated using the dialysis bag diffusion method. Artificial saliva (pH = 6.5–7.0) was employed as the release medium and added to 50 mL stoppered conical flasks. One milliliter of each sample solution was transferred into a dialysis bag, which was then immersed in the conical flask and incubated at 37 °C with shaking at 120 rpm. At predetermined time intervals (0, 10, 20, 30, 40, 50, 60, 70, and 80 h), 3 mL of the external release medium was collected and immediately replaced with an equal volume of pre-warmed fresh artificial saliva. The optical density (OD) at each time point was determined using a UV-visible spectrophotometer. All experiments were carried out in triplicate, and the mean values were calculated. The cumulative drug release percentages at different time intervals were obtained to characterize the *in vitro* release profile of CEN composites.

### 2.3 Remineralization experiment on demineralized enamel

#### 2.3.1 Preparation of demineralized enamel specimens

One hundred human premolars with intact enamel (free of caries, cracks, or defects) extracted for orthodontic reasons were selected. Soft tissues, calculus, and pigments were removed from the tooth surfaces. After mechanical cleaning, the buccal crowns were sectioned into enamel blocks of 4 × 3 × 2 mm^3^ using a diamond bur. The specimens were rinsed with 75% ethanol and stored in 0.1% thymol solution at 4 °C until further use. The enamel blocks were etched with 37% phosphoric acid for 30 s to establish a demineralized model, then rinsed with deionized water and cleaned ultrasonically for 5 min, followed by storage at 4 °C (Shao et al., 2019).

#### 2.3.2 Screening of the optimal CEN composite concentration

Seventy demineralized enamel blocks were randomly and equally divided into 7 groups: 6 experimental groups as described in Section 2.1.3, and an artificial saliva control group.

After gentle rinsing and drying, 10 specimens from each group were immersed in the corresponding treatment solutions for 6 h, rinsed with deionized water, and incubated in artificial saliva at 37 °C for 18 h. This cycle was repeated daily for 7 consecutive days, yielding a total remineralization period of 42 h (Wen et al., 2024). Upon completion of the experiment, the specimens were thoroughly rinsed with deionized water and dried at 25 °C.

Microhardness testing was conducted before and after remineralization using an HMV-2T Vickers microhardness tester with a 100 g load and a 15 s dwell time. Three points were measured on each surface, and the mean value was recorded as the surface microhardness. Scanning electron microscopy (SEM) coupled with energy-dispersive X-ray spectroscopy (EDS) was employed to characterize the surface morphology and elemental composition of the experimental samples at 1, 3, and 7 days following remineralization treatment, based on which the CEN complex with the optimal remineralization performance was identified.

#### 2.3.3 Remineralization efficacy of the optimal CEN composite

Thirty demineralized enamel blocks were randomly and equally divided into 3 groups (n = 10): Control group: artificial saliva; CS-CaP group; Optimal CEN composite group. The control group was immersed in artificial saliva continuously, with fresh medium replaced daily. Specimens in the CS-CaP and optimal CEN groups were immersed in treatment solutions for 6 h daily, rinsed, and incubated in artificial saliva at 37 °C for 18 h for 7 consecutive days (total remineralization time: 42 h). All specimens were rinsed and dried as previously described.

Vickers microhardness was measured before and after remineralization. SEM coupled with EDS was employed to characterize the surface morphology and elemental composition of the experimental samples at 1, 3, and 7 days following remineralization treatment.

### 2.4 Cytotoxicity assay

#### 2.4.1 Cell culture

Human Oral Keratinocytes (HOK) at passages 10–15 were used.Cells were cultured in DMEM medium supplemented with 10% fetal bovine serum (FBS) in a humidified incubator at 37 °C with 5% CO_2_. The medium was refreshed every other day. When cells reached approximately 80% confluence, they were digested with 0.25% trypsin and subcultured.

#### 2.4.2 Cell counting Kit-8 (CCK-8) assay

HOK cells were used to evaluate the cytotoxicity of CEN composites. Cells were cultured in DMEM containing 10% FBS, 100 U/mL penicillin, and 100 μg/mL streptomycin at 37 °C with 5% CO_2_. The average subculture cycle was 2–3 days.

Cells were divided into a blank control group (DMEM medium) and an optimal CEN concentration group, with three replicate wells per group. HOK cells in the logarithmic growth phase were digested, counted, and seeded into 96-well plates at a density of 5 × 10^3^ cells/well and cultured for 24 h. After cell adhesion, the medium was replaced with medium containing CEN solution diluted at ratios of 1/1, 1/10, 1/20, 1/50, and 1/100 (Tomaz et al., 2020).

Cells were incubated for another 24, 48, and 72 h. Subsequently, 10 μL CCK-8 solution was added to each well in the dark, mixed gently without bubbling, and incubated for 1 h. Absorbance at 450 nm was measured using a microplate reader to assess cell proliferation activity.

$$\text{Cell viability }\left(\%\right)=\left(A_{\text{experimental}\;\text{group}}-A_{\text{blank}\;\text{group}}\right)/\times \left(A_{\text{experiental}\;\text{group}}-A_{\text{blank}\;\text{group}}\right)\times 100\%$$

## 3 Experimental results

### 3.1 Material characterization

**FIGURE 1:**
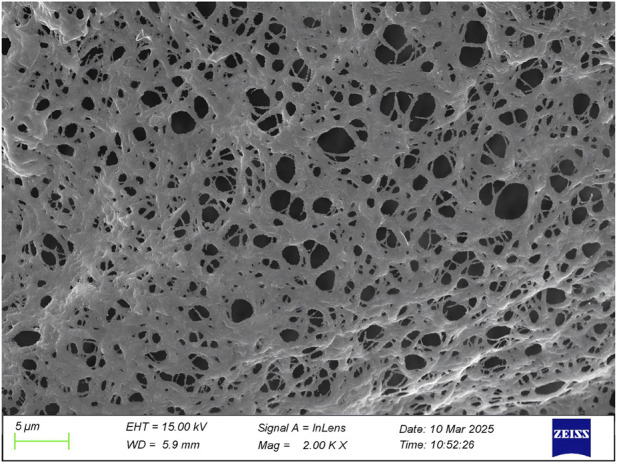
1.5% CS-CaP electron microscope.

**FIGURE 2:**
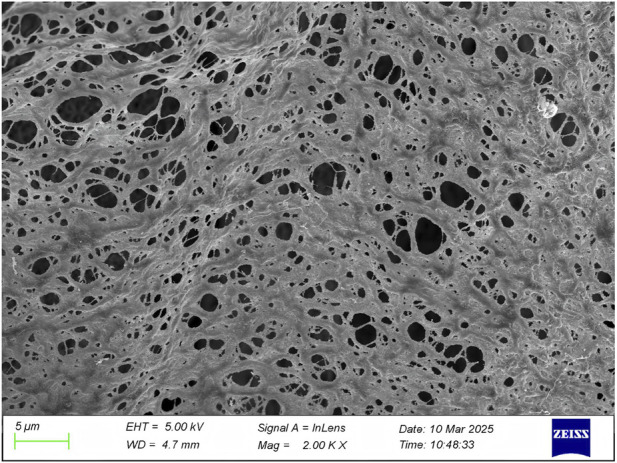
2% CS-CaP electron microscope.

**FIGURE 3:**
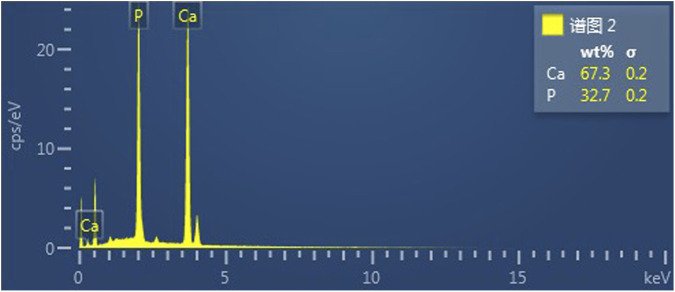
EDS results for 1.5% CS-CaP.

**FIGURE 4:**
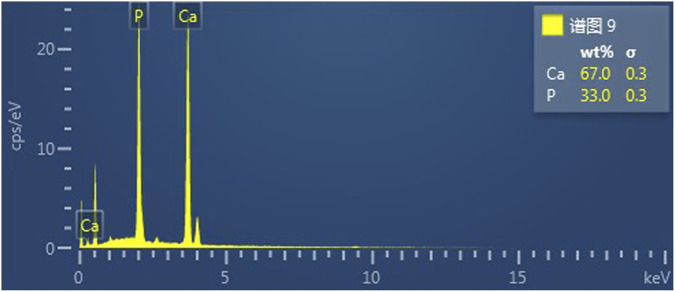
EDS results for 2% CS-CaP.

**FIGURE 5:**
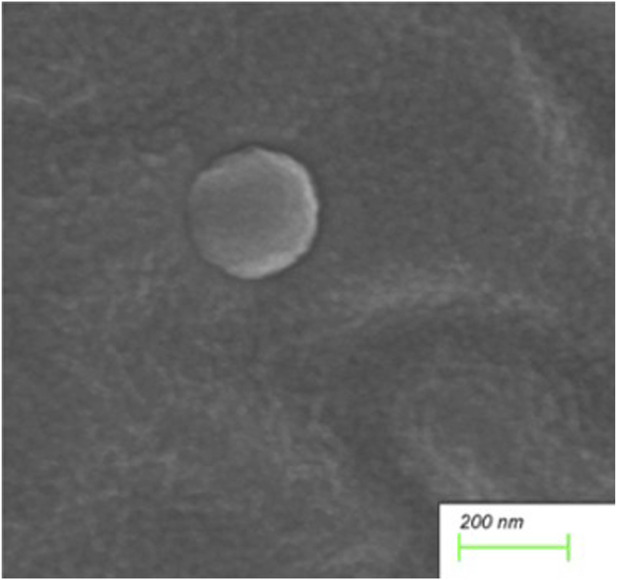
Electron micrograph of EGCG-NPs particle size distribution.

**FIGURE 6:**
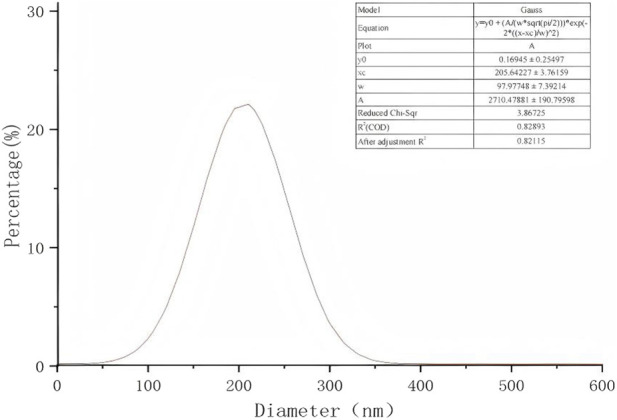
Particle size distribution of EGCG-NPs.

Scanning electron microscopy revealed the presence of three-dimensional network structures in 1.5% and 2% chitosan-calcium phosphate gels (Figures 1, 2). Energy-dispersive X-ray spectroscopy showed that the calcium/phosphorus molar ratio of the hydrogel was similar to that of natural enamel (1.67) (Figures 3, 4). Particle size analysis of EGCG-NPs demonstrated uniformly spherical nanoparticles with an average diameter of 205 nm (Figures 5, 6). Zeta potential measurements yielded a value of approximately −25.63 mV for EGCG-NPs. The drug loading efficiency was calculated as 0.633/10% × 100% = 6.33%, and the encapsulation efficiency was determined as: drug loading efficiency/drug-to-carrier mass ratio = 75.9%.

**FIGURE 7:**
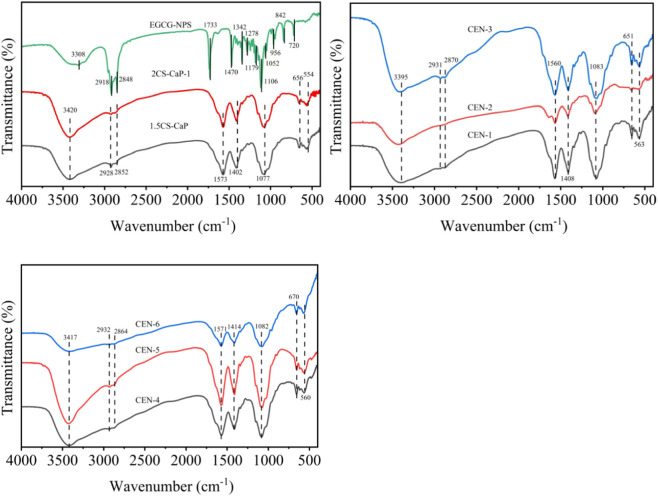
Fourier transform infrared spectra.

Fourier transform infrared spectroscopy characteristic peak analysis (Figure 7): The broad peak at 3,308 cm^-1^ can be attributed to the superimposed stretching vibrations of the phenolic hydroxyl (O-H) groups in EGCG and the N-H groups in soybean lecithin. The increased peak width suggests the formation of strong hydrogen-bonding interactions between EGCG and the lipid matrix. The double peaks at 2,918 and 2,848 cm^-1^ arise from the symmetric and antisymmetric C-H stretching vibrations of the alkyl chains in glycerol monostearate and stearic acid, confirming the successful formation of the lipid matrix. The strong peak at 1,733 cm^-1^ is assigned to the stretching vibration of the ester carbonyl (C=O) group in glycerol monostearate. The disappearance of the characteristic carbonyl peak of gallic acid (approximately 1,690 cm^-1^) present in free EGCG indicates that EGCG has been effectively encapsulated within the lipid core. Identical absorption peaks were observed at 3,420, 2,928, 2,852, 1,573, 1,402, 1,077, 656, and 554 cm^-1^ in both the 1.5% and 2% CS-CaP samples, verifying the successful synthesis of the chitosan-calcium phosphate composite. The broad peak at 3,420 cm^-1^ can be assigned to the superimposed O-H and N-H stretching vibrations within the chitosan molecule. The relatively narrower peak width at this position in the 2% CS-CaP sample may be related to the enhanced molecular chain packing and strengthened hydrogen bonding caused by the higher chitosan concentration (0.15–0.20 g), as well as the changed intermolecular forces due to the increased viscosity of the higher-concentration chitosan solution. CEN-4-6 (chitosan concentration 2%) and CEN-1-3 (chitosan concentration 1.5%) exhibited nearly identical characteristic peak positions at 3,417 cm^-1^ (corresponding to O-H/N-H stretching vibration), 2,932 cm^-1^ (asymmetric C-H stretching vibration), and 1,082 cm^-1^ (C-O-C glycosidic bond stretching vibration). This indicates that the main-chain structure of chitosan and its binding mode with EGCG nanoparticles remained relatively stable within this concentration range. However, the peak at 3,417 cm^-1^ in sample CEN-4-6 showed a slight blue-shift compared with the peak at 3,395 cm^-1^ in sample CEN-1-3. This shift can be attributed to the enhanced intermolecular hydrogen-bonding networks caused by the increased chitosan concentration (2%). The strengthened hydrogen-bonding interactions between the hydroxyl and amino groups of chitosan and the phenolic hydroxyl groups of EGCG resulted in the broadening of the O-H/N-H vibrational energy distribution, as reflected by the widened peak. Meanwhile, the higher EGCG concentration in sample CEN-6 may promote the formation of denser complexes via electrostatic attraction or hydrophobic interactions between phenolic hydroxyl groups and chitosan. This further alters the local microenvironment and causes the O-H vibration frequency to shift toward higher wavenumbers. The stable occurrence of the C-O-C bond at 1,082 cm^-1^ confirms that the glycosidic bonds of chitosan remain intact during the complexation process. The consistent detection of PO_4_^3-^ lattice vibration peaks at 651 and 563 cm^-1^ indicates that the aromatic moieties of EGCG nanoparticles are stably combined with chitosan through π-π stacking or coordination interactions.

**FIGURE 8:**
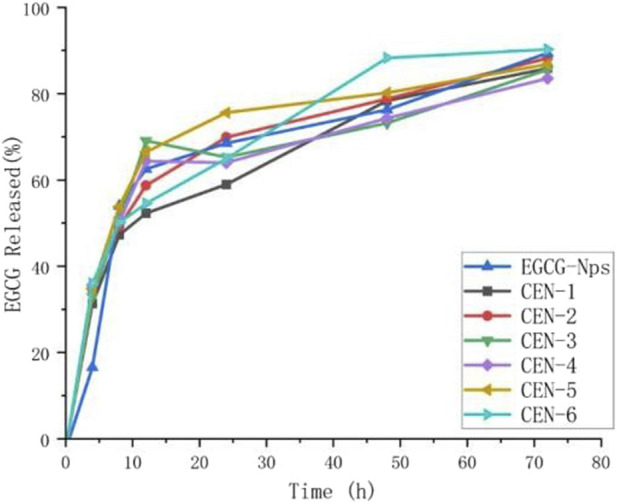
Release rate of EGCG nanoparticles.

Line graph showing the percentage of EGCG released over time for seven sample types, including EGCG -Nps and CEN-1 to CEN-6, each represented by distinct colored lines. Time in hours is plotted on the x-axis and EGCG released percentage on the y-axis. All samples show a rapid initial increase, then gradual rise to around 80-90 percent release over seventy-two hours. Legend identifies lines by color and symbol (Figure 8).

### 3.2 Screening results for the optimal concentration of the CEN complex

**FIGURE 9:**
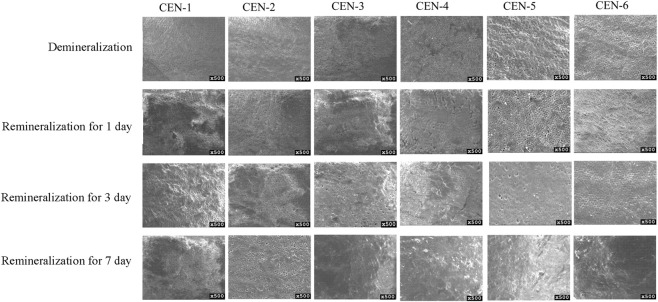
CEN composite remineralisation experiment electron microscope.

Scanning electron microscopy observations (Figure 9) showed that after demineralization, the surface structure of tooth enamel was severely damaged, with substantial loss of calcium, phosphorus, and other minerals. Numerous irregular pores and pit-like defects were observed on the surface, representing typical microstructural characteristics caused by acid-induced dissolution of enamel crystals. After treatment with the CEN composite, remineralized deposits were formed on the enamel surface; the number and size of pores were significantly reduced, and pores were almost completely eliminated in some regions, leading to partial restoration of the enamel surface. As shown in Figure 9, although the enamel surfaces in the CEN-1, CEN-2, and CEN-3 groups were improved compared with the demineralized group, with decreased pore diameters, scattered small pits and micropores were still visible, indicating relatively limited remineralization. In contrast, the enamel surfaces in the CEN-4, CEN-5, and CEN-6 groups were smoother and denser, with no obvious pores or pit structures, demonstrating significantly more effective remineralization. In summary, the CEN composite can more effectively promote the deposition of calcium, phosphorus, and other ions on the demineralized enamel surface, thereby repairing acid-induced enamel defects and exhibiting favorable remineralization efficacy. The remineralization and repair effects on demineralized enamel were significantly more pronounced in the CEN-4, CEN-5, and CEN-6 groups than in the CEN-1, CEN-2, and CEN-3 groups.

**FIGURE 10:**
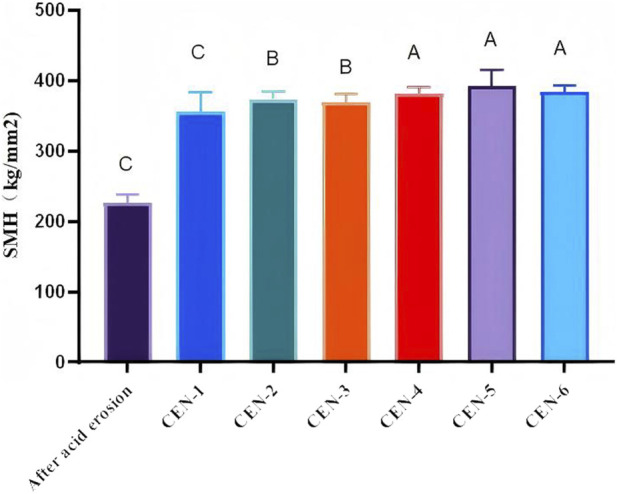
Enamel Microhardness Results. Two-way comparisons between groups ***<0.001.

Micro-Vickers hardness of demineralized and remineralized dental enamel was evaluated. Data were analyzed using one-way analysis of variance (ANOVA) for pairwise intergroup comparisons, and 95% confidence intervals (CI) were calculated. The demineralized group exhibited a mean microhardness of 226.31 ± 11.24 HV. Compared with the demineralized group, all CEN experimental groups showed a significant increase in microhardness after remineralization (all P < 0.001), and none of the 95% CIs crossed zero, confirming the statistical reliability (Figure 10). Intergroup differences were statistically significant (P < 0.05; Table 1), among which the CEN-5 group presented the highest micro-Vickers hardness value following remineralization.

**TABLE 1:** Results of micro-vickers hardness testing on enamel before and after remineralisation.

Group	n	SMH	F	P
After acid etching	10	226.31 ± 11.44	5.85	<0.001
CEN-1	10	355.41 ± 27.24		
CEN-2	10	373.80 ± 11.33		
CEN-3	10	369.77 ± 11.05		
CEN-4	10	381.25 ± 9.95		
CEN-5	10	392.48 ± 23.16		
CEN-6	10	383.64 ± 9.12		

Energy-dispersive X-ray spectroscopy (EDS) was performed to investigate changes in the Ca/P molar ratio of remineralized enamel layers across groups after 7 days of cyclic immersion in experimental solutions and artificial saliva (Table 2). The results demonstrated that the Ca/P molar ratio displayed an overall upward trend over time in all groups, indicative of mineral deposition to varying degrees. Following 7 days of treatment, the Ca/P molar ratio of enamel in each calcium-enriched nanocomplex (CEN) group was significantly elevated relative to acid-etched enamel (Ca/P molar ratio = 1.18). Notably, the CEN-5 group displayed a Ca/P molar ratio most consistent with stoichiometric hydroxyapatite (Ca/P molar ratio = 1.67).

**TABLE 2:** EDS test results for enamel before and after remineralization.

Element	After acid etching	CEN-1	CEN-2	CEN-3	CEN-4	CEN-5	CEN-6
Ca/P	1.18	1.48	1.53	1.56	1.57	1.62	1.54

Based on the integrated analysis of scanning electron microscopy (SEM) observations, micro-Vickers hardness data, and EDS findings, the CEN-5 group was confirmed to achieve the optimal remineralization efficacy.

### 3.3 Results of CEN complex remineralisation experiments

**FIGURE 11:**
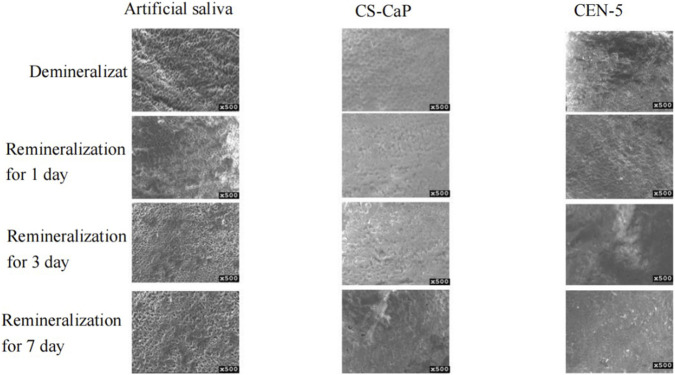
Electron microscopy of CEN composite remineralisation experiments.

In this study, an *in vitro* artificial saliva system was employed to simulate the oral microenvironment, and remineralization experiments were conducted on demineralized dental enamel to compare the remineralization efficacy of different experimental solutions. Scanning electron microscopy (SEM) was utilized to observe the surface morphological characteristics of enamel samples from various groups after cyclic immersion in experimental solutions and artificial saliva (Figure 11). The results revealed that in the control group, where demineralized enamel was immersed in artificial saliva alone, nascent mineral deposition was observed on the enamel surface, and the deposition amount showed an increasing trend with prolonged immersion time. By day 7, a thin mineral layer had formed on the enamel surface; however, the characteristic fish-scale structure was still visible beneath this layer. This finding indicates that relying solely on the low concentrations of calcium and phosphate ions provided by artificial saliva is insufficient to achieve efficient remineralization of demineralized enamel, manifesting obvious inefficiency in the mineralization process. Compared with the control group, the CS-CaP group exhibited superior mineralization efficacy at all time points (1, 3, and 7 days of immersion), with the surface pore structures becoming shallower and concurrent deposition of mineral particles; nevertheless, the particles were unevenly distributed and loosely structured. In contrast, the demineralized enamel in the CEN group displayed a more rapid remineralization process, with continuous accumulation of mineral deposits over time. By day 7, the smoothness of the enamel surface was significantly improved, and the surface pores and defect structures were almost completely eliminated compared with those on day 1. By comparing the enamel morphology between the CEN group and the CS-CaP group, it was concluded that the complex containing EGCG-NPs exerted more remarkable remineralization effects than the CS-CaP alone group, demonstrating that EGCG-NPs facilitate the remineralization of dental enamel.

**FIGURE 12:**
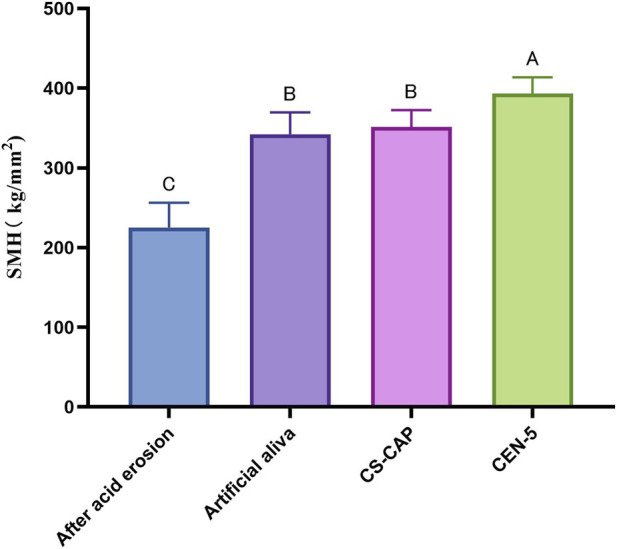
Microhardness results of enamel. Intergroup comparison *** <0.001.

Micro-Vickers hardness was evaluated in demineralized enamel and in enamel treated with artificial saliva, CS-CaP, or CEN-5. Microhardness data were analyzed using one-way analysis of variance (ANOVA) for multiple comparisons, with 95% confidence intervals (CIs) calculated to assess statistical reliability. The demineralized group exhibited a mean microhardness of 225.25 ± 31.04 HV. Compared with the demineralized group, all experimental groups showed a significant increase in microhardness following remineralization (all P < 0.001; Figure 12), and all 95% CIs did not cross zero, confirming the statistical robustness of the findings. Statistically significant differences were observed among groups (P < 0.05; Table 3). The ranking of hardness values revealed the following order: CEN-5 > CS-CaP > artificial saliva > demineralized group.

**TABLE 3:** Micro vickers hardness test results of enamel before and after remineralization.

Group	n	SMH	F	P
After acid erosion	10	225.25 ± 31.04	13.74	<0.001
Artificial saliva	10	338.13 ± 29.38		
CS-CaP	10	346.41 ± 25.86		
CEN-5	10	384.54 ± 34.91		

EDS Results: EDS analysis unveiled alterations in the calcium-to-phosphorus (Ca/P) molar ratio of the remineralized enamel layer in distinct groups subsequent to 7 days of cyclic immersion in experimental solutions and artificial saliva (refer to Table 4). The findings suggested that the Ca/P molar ratio generally ascended over time across all groups, signifying varying extents of mineral deposition. After a seven - day period, the Ca/P molar ratio of enamel treated with each solution group had significantly elevated in comparison to acid - etched enamel (Ca/P molar ratio = 1.12). The CEN-5 group presented a Ca/P molar ratio most consistent with stoichiometric hydroxyapatite (Ca/P molar ratio = 1.67).

**TABLE 4:** EDS test results for dental enamel before and after remineralisation.

Element	After acid erosion	Artificial saliva	CS-CaP	CEN-5
Ca/P	1.12	1.32	1.46	1.60

In conclusion, the CEN group exhibited notably superior remineralization compared to the artificial saliva and CS - CaP groups, which validates that the incorporation of EGCG - NPs substantially enhances the remineralization efficacy.

### 3.4 CCK - 8 assay results

**FIGURE 13:**
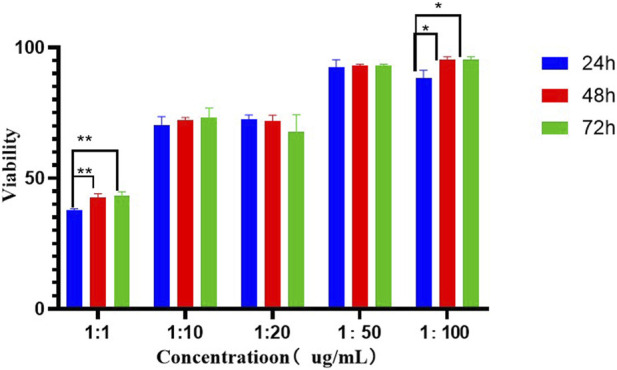
CCK-8 assay for cell viability.

Cell viability was evaluated via the CCK - 8 assay. As depicted in Figure 13, the CEN group demonstrated cell viability exceeding 75% at dilutions of 1/20, 1/50, and 1/100 of the culture medium. In accordance with the cytotoxicity classification criteria stipulated in ISO 10993–5:2009, materials with cell survival rates surpassing 75% are classified as non - cytotoxic. These results clearly confirm that the CEN complex at this concentration shows no significant cytotoxicity.

## 4 Discussion

Chitosan, as a natural polymer material, is widely utilized as an organic matrix for remineralization due to its excellent biocompatibility, biodegradability, and ease of chemical modification. By incorporating nanofunctional materials, these hydrogels can simultaneously fulfill two core functions: serving as an “ion reservoir” for the sustained supply of calcium and phosphate ions, and providing a high density of heterogeneous nucleation sites. This synergistic effect significantly enhances the density and surface hardness of the enamel remineralization layer (Liao et al., 2024). Recent studies have further expanded the applications in this field. Incorporating mesoporous bioactive glass (MBG) into chitosan hydrogels leverages its high specific surface area and mesoporous structure to efficiently load and slowly release fluoride ions, achieving sustained enamel remineralization for up to 5 days, with the hardness of the restored enamel even recovering to above-normal levels (Yang L. et al., 2025). Furthermore, chitosan hydrogels constructed via self-assembly of amelogenin-derived peptides (e.g., P26), known as P26-CS, exhibit excellent biocompatibility and structural stability. They can penetrate deep into carious white spot lesions and induce the ordered deposition of needle-like mineral crystals, thereby enabling the layered repair of both enamel and dentin (Dogan et al., 2018).

Epigallocatechin gallate (EGCG), the primary bioactive tea polyphenol in green tea, has seen its mechanism of action in oral hard tissue protection expand from a simple antibacterial effect to multifunctional regulation. Research indicates that EGCG can not only reduce the acid-producing capacity of dental plaque by inhibiting bacterial dehydrogenase activity, thereby maintaining the local pH balance on the enamel surface and effectively preventing the initiation and progression of dental caries (Ye and Wang, 2025). In terms of dentin protection, flavonoid compounds, with EGCG as a key representative, demonstrate significant potential: they can effectively inhibit the activity of matrix metalloproteinases (MMPs), protecting exposed collagen fibrils from degradation. Additionally, they can form hydrogen bonds with collagen fibrils through their polyphenolic hydroxyl groups, stabilizing the collagen network structure and providing a scaffold for subsequent mineralization. Studies have shown that the combined effect of EGCG in reducing overall tooth structure loss and promoting remineralization may even be superior to traditionally used fluoride and chlorhexidine (Nunes et al., 2025).

More in-depth mechanistic studies have revealed the direct regulatory role of EGCG on the mineralization activity of host cells. Experiments have confirmed that EGCG can modulate the secretory activity of odontoblast-like cells, significantly upregulate alkaline phosphatase (ALP) activity, and accelerate the formation and deposition of mineralized nodules. This indicates its potential value in promoting reparative dentin formation and dentin mineralization repair (Duque et al., 2025).

In this study, epigallocatechin gallate (EGCG) was prepared into nanoparticles (EGCG-NPs) and uniformly dispersed within a chitosan-calcium phosphate (CS-CaP) gel. A dual-functional system integrating sustained drug release and a mineralization template was thereby constructed, aiming to promote remineralization deposition on demineralized enamel surfaces and repair enamel white spot lesions. Through *in vitro* remineralization assays, the optimal concentration of the CEN (CS/CaP/EGCG-NPs) composite was identified. Subsequent experiments confirmed that the incorporation of EGCG-NPs significantly enhanced the remineralization efficacy. The microhardness of the enamel was significantly enhanced after remineralization, with its Ca/P ratio most consistent with stoichiometric hydroxyapatite. Results from the CCK-8 assay demonstrated that the cell viability remained above 70% in the culture media containing the CEN complex at dilutions of 1:10, 1:20, 1:50, and 1:100, indicating that the CEN complex is non-cytotoxic. Material characterization further confirmed stable chemical interactions among the constituent components.

In conclusion, the CS-CaP gel loaded with EGCG-NPs achieves the repair of enamel white spot lesions through a synergistic mechanism. The prominent advantage of this system lies in the complementary functions of its components: CS can effectively chelate calcium ions (Ca^2+^), while CaP serves as a stable ion reservoir. Additionally, EGCG-NPs exert a synergistic effect to efficiently promote enamel remineralization.

## 5 Prospects and limitations

However, the remineralization effect of the materials prepared in this study has only been verified under *in vitro* conditions. *In vitro* experiments cannot fully simulate the actual oral environment. Therefore, future studies should further optimize the enamel white spot lesion model, and systematically evaluate its long-term *in vivo* stability, remineralization capacity, and biocompatibility. Additional comparisons with clinically established remineralizing agents are warranted to enhance its potential for clinical translation.

## Data Availability

The original contributions presented in the study are included in the article/supplementary material, further inquiries can be directed to the corresponding author.
